# Relating solvatochromism and solvatomorphism in organic dyes using high pressure

**DOI:** 10.1107/S2052252524005773

**Published:** 2024-06-27

**Authors:** Elena Boldyreva

**Affiliations:** a Novosibirsk State University ul Pirogova 2 Novosibirsk 90 Russian Federation

**Keywords:** high pressure, solvatomorphism, solvatochromism, piezochromism, intermolecular interactions, supramolecular associates, crystal solvates

## Abstract

By using complementary experimental methods including *in situ* high-pressure single-crystal X-ray diffraction and UV–Vis spectroscopy, the intricate connection between solvatochromism and solvatomorphism has been elucidated in a recent publication [Sobczak & Katrusiak (2024). *IUCrJ*, **11**, 528–537]. The connection was demonstrated for an important pigment – Reichardt’s dye – with potential applications in nonlinear optoelectronics and molecular pressure sensor development.

I remember my early childhood experience with ‘secret ink’ that only became visible when a sheet of paper was sprayed with ethanol. This was a manifestation of *solvatochromism*, color change in different solvents (Marini *et al.*, 2010 ▸). Cobalt chloride hexahydrate is pale pink and any text made using its aqueous solution is hardly visible on a white or pinkish background. However, once cobalt chloride is dissolved in ethanol or dried, the resulting dehydrated form is blue, so that the text becomes clearly visible. Later I learnt that the color difference is related to the coordination of cobalt (octahedral in the hydrate and tetrahedral in the anhydrous form that is formed on contact with ethanol) and to the ligand field splitting of the orbitals (Griffith & Orgel, 1957 ▸).

Fascinating color change of solutions of transition metal salts in different solvents is one of the main attractions to the world of chemistry for many children. Organic compounds are also known to form solutions of different colors when dissolved in different solvents. This is due to different supramolecular associates formed by a dissolved molecule (solute) and the solvent molecules. Solvatochromism is commonly used in many areas of chemical and biological research to study bulk and local polarity in macrosystems (membranes *etc*.), or even the conformation and binding of proteins.

Despite its wide use, solvatochromism remains a largely unexplored phenomenon due to the extremely complex coupling of the many different interactions and dynamic processes that characterize it.

It is difficult to get direct information on the environment of a chromophore in solution. This challenge is exacerbated by the dynamics of the environment itself, which is labile and versatile in solution, in contrast to the solid state. Crystal structures of crystal solvates shed some light on the relation between the immediate environment of a chromophore in the condensed state and the optical spectrum that defines the observed color. Therefore, crystallization from different solvents can be used to model the solvation process, at least to some extent.

Pressure can fine tune the interatomic distances with or without changing the general symmetry and geometry of coordination; in this way optical properties can also be fine-tuned. The phenomenon is known as *piezochromism* (Reichardt, 1992 ▸). The mechanisms of piezochromism may be related to molecular rearrangement, conformational changes, breaking chemical bonds, or multiple cooperative phenomena (Li *et al.*, 2021 ▸). In crystals, structural changes can be followed directly by diffraction techniques and then related to changes in optical properties measured spectroscopically. It is more difficult to get such information for a molecular environment in solution.

In an article in this issue of **IUCrJ** (Sobczak & Katrusiak, 2024 ▸), the techniques of high-pressure crystallization, *in situ*X-ray diffraction and UV–vis spectroscopy were combined to understand structural aspects of solvatochromism. The authors focused their attention on a representative of a large class of the *N*-betaine dyes, Reichardt’s dyes, namely 4-(2,4,6-tri­phenyl-1-pyridinio)phenolate [ET(1)]. These dyes are particularly sensitive to changes in the solvent environment and are thus used as probes for studying solvent–solute interactions (Reichardt, 1994 ▸). Their molecules comprise an extended conjugated and polarizable system, with a large permanent dipole moment. Additionally, the oxygen atom on the phenolate moiety is highly basic and prone to form hydrogen bonds with solvent molecules. The solvatochromic effect in this group of compounds was postulated to originate from differences between solvation spheres formed around the dye’s highly dipolar electronic ground state and its less dipolar excited state (Reichardt, 1994 ▸).

The ET(1) crystals were put into a diamond anvil cell (DAC) immersed in a solution of ET(1) in a selected solvent (acetone, methanol or ethanol) and then compressed. Optical spectra of the solutions were measured as a function of pressure. At certain pressures, the original ET(1) crystals were found to recrystallize into solvates, and could be prepared *in situ* as single crystals at high pressure, facilitating their structure determination by single-crystal X-ray diffraction (Fig. 1 ▸).

A detailed analysis of the chromophore environment in the solid state helped shed light on the relation between molecular structure, solute–solvent interactions and optical properties. By comparing the optical spectra in the solid state and in solution, the authors used this information to indirectly probe solution structure. Interestingly, on approaching crystallization, there were anomalous changes in the pressure dependence of the absorbance spectra, indicating clearly that while the zwitterionic form of the ET(1) was stabilized in the solvation sphere, the interactions formed with the solvent molecules were significantly altered above the hydro­static limit of the solvent.

Relations between *solvatochromism* and *piezochromism* have attracted significant attention for a long time. Research has usually dealt with the effects of pressure on solutions of piezochromic compounds in different solvents (Burgess *et al.*, 1998 ▸). However, solvents may also have a pronounced effect on the piezochromism of solids. To achieve hydro­static compression, solids are not studied at high pressure in isolation, but are immersed in a pressure-transmitting fluid. This fluid can interact with the solid and result in high-pressure solvate formation, solvent-assisted polymorphic transitions, or recrystallization (Zakharov & Boldyreva, 2019 ▸). One could expect that a pressure-transmitting fluid may also affect the piezochromism of crystals under hydro­static compression. This phenomenon has not been studied yet though one can consider the recent contribution by Sobczak & Katrusiak (2024 ▸) as the first step in this direction. The pressure-transmitting medium in their work affected solid-state piezochromism *via* the formation of different solvates. One can also expect that other mechanisms are possible for other compounds, perhaps related to surface solid–liquid interactions or liquid-assisted recrystallization into other polymorphs (not a solvate). Studies of solvatochromic shifts under varying pressure conditions are challenging, but, as the recent work by Sobczak & Katrusiak illustrates so clearly, they open new avenues for understanding solvent-mediated effects in molecular systems. They also pave the way for exploring potential applications in pressure-sensitive molecular sensors and materials science, particularly in the development of advanced photonic materials.

## Figures and Tables

**Figure 1:**
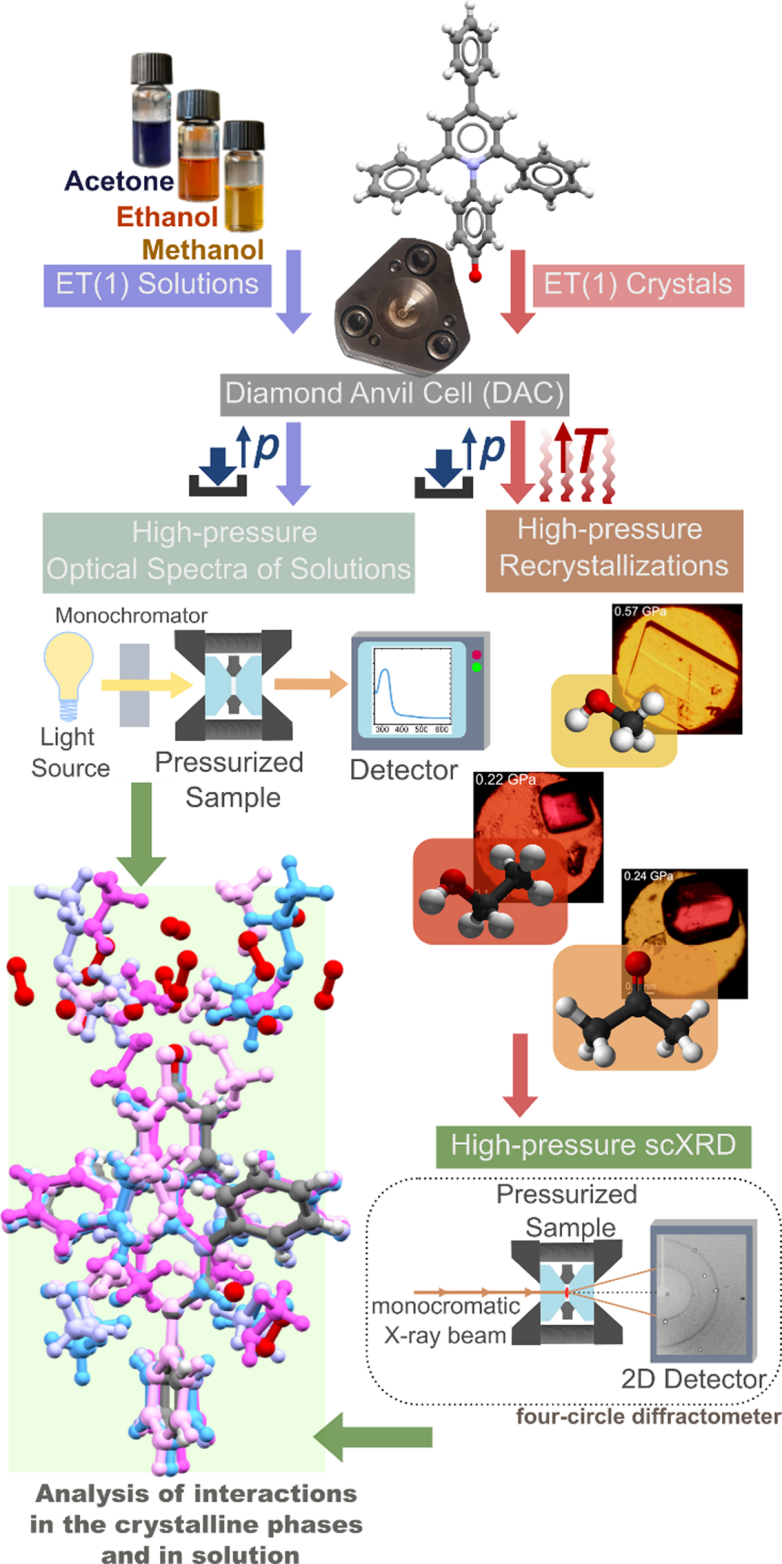
Schematic overview of experimental procedures from Sobczak & Katrusiak (2024 ▸).
